# Supplementary material for: Serological Evidence of Crimean–Congo Haemorrhagic Fever in Livestock in the Omaheke Region of Namibia
Source: Microorganisms. 2024 Apr 22;12(4):838. doi: 10.3390/microorganisms12040838 (PMC11051821; doi:10.3390/microorganisms12040838)
Supplement: Supplementary file 1 [file microorganisms-12-00838-s001.zip › Supplementary Table S1.pdf]

Table S1: A summary table displaying ELISA test results for CCHF conducted on 100 cattle from 24 farms sampled at an auction gathering.

Farm No.	No. sampled	No. positive	% Positive
1	4	2	50
2	4	3	75
3	4	3	75
4	1	0	0
5	5	4	80
6	1	1	100
7	3	3	100
8	2	1	50
9	2	2	100
10	1	1	100
11	10	8	80
12	2	0	0
13	3	0	0
14	7	1	14
15	1	0	0
16	3	1	33
17	5	2	40
18	28	3	11
19	7	1	14
20	3	0	0
21	1	0	0
22	1	0	0
23	1	0	0
24	1	0	0
TOTAL	100	36	36
