# Supplementary material for: Serological Evidence of Crimean–Congo Haemorrhagic Fever in Livestock in the Omaheke Region of Namibia
Source: Microorganisms. 2024 Apr 22;12(4):838. doi: 10.3390/microorganisms12040838 (PMC11051821; doi:10.3390/microorganisms12040838)
Supplement: Supplementary file 1 [file microorganisms-12-00838-s001.zip › Supplementary Table S2.pdf]

Table S2: A summary table depicting the ELISA test results for CCHF in sheep sampled from 22 farms at an auction.

Farm No.	No. sampled	No. positive	% Positive
1	2	0	0
2	7	0	0
3	4	0	0
4	10	1	10
5	3	0	0
6	12	9	75
7	1	1	100
8	1	0	0
9	5	3	60
10	16	2	13
11	3	1	33
12	7	1	14
13	5	0	0
14	3	0	0
15	2	0	0
16	1	0	0
17	4	1	25
18	3	1	33
19	6	2	33
20	3	0	0
21	1	0	0
22	1	0	0
TOTAL	100	22	22
